# Reflection on the Challenges, Accomplishments, and New Frontiers of Gene Drives

**DOI:** 10.34133/2022/9853416

**Published:** 2022-08-06

**Authors:** Michael Melesse Vergara, Jesse Labbé, Joanna Tannous

**Affiliations:** ^1^Biosciences Division, Oak Ridge National Laboratory, Oak Ridge, TN 37831, USA; ^2^Invaio Sciences, Cambridge, MA 02138USA

## Abstract

Ongoing pest and disease outbreaks pose a serious threat to human, crop, and animal lives, emphasizing the need for constant genetic discoveries that could serve as mitigation strategies. Gene drives are genetic engineering approaches discovered decades ago that may allow quick, super-Mendelian dissemination of genetic modifications in wild populations, offering hopes for medicine, agriculture, and ecology in combating diseases. Following its first discovery, several naturally occurring selfish genetic elements were identified and several gene drive mechanisms that could attain relatively high threshold population replacement have been proposed. This review provides a comprehensive overview of the recent advances in gene drive research with a particular emphasis on CRISPR-Cas gene drives, the technology that has revolutionized the process of genome engineering. Herein, we discuss the benefits and caveats of this technology and place it within the context of natural gene drives discovered to date and various synthetic drives engineered. Later, we elaborate on the strategies for designing synthetic drive systems to address resistance issues and prevent them from altering the entire wild populations. Lastly, we highlight the major applications of synthetic CRISPR-based gene drives in different living organisms, including plants, animals, and microorganisms.

## 1. Introduction

A population’s allele frequency often obeys Mendelian inheritance patterns due to independent allele assortment. However, deviations from these norms are commonly encountered in nature. Selfish genetic elements are DNA fragments that can control and enhance their own intergenerational transmission in greater than Mendelian frequencies, at the expense of other alleles. This results in population-wide genetic changes, even if negative impacts on organismal fitness were encountered [1, 2]. A number of naturally occurring selfish genetic elements have been discovered to date [3] and served as the foundation for synthetic gene drives that hold potential for spreading engineered traits through wild populations [4, 5].

So far, synthetic gene drives have been established with inconsistent success rates in many living organisms, including mosquitoes, flies, some groups of microbes, and more recently in plants [6–10]. However, these systems have only been tested and explored in a laboratory setting; thus, how they will perform in natural environments remains unclear. Like conventional biocontrol agents, gene drive organisms could establish themselves in the environment and spread [11, 12]. This self-propagating nature of gene drives might result in possible benefits but can also pose significant risks. On the one hand, this technique could help addressing several serious universal challenges, including the spread of microbial infections and diseases carried and transmitted by vectors, the upsurge of resistance to pesticides, and the agricultural and ecological harms associated with invasive species [13]. Yet, on the other hand, releasing gene drive-modified organisms into the environment increases the challenge of their containment if unintended effects occurred, or if they spread into nontarget populations [14]. Therefore, for the benefits of this technology to be achieved, drive systems should be engineered to be efficient at spreading effectively into populations to attain their anticipated impact while being able to be recalled in the occurrence of undesirable consequences or public disfavor. A recent survey conducted in California showed a strong rejection for the use of gene drive technology as many respondents expressed serious concerns including undesired environmental outcomes, impacts on human health, and high expenses [15].

The emergence of the adaptive bacterial immunity system “clustered regularly interspaced short palindromic repeats/associated proteins” (CRISPR-Cas) has revolutionized and democratized the genome engineering field. This naturally occurring system works by memorizing foreign DNAs in unique spacer sequences. Once new attacks from familiar encounters occur, the transcripts of the CRISPR spacers are used to recognize homogenous sequences and direct Cas nucleases to disrupt and inactivate the target sites [16]. This groundbreaking discovery and the extensive use of Cas endonucleases as genome-editing tools have made the engineering of gene drive systems so much easier and highly efficient [13, 17]. This paper comprehensively reviews the natural forms of gene drive discovered to date and how these principles were leveraged into synthetic approaches engineered to address ecological and medical concerns. We later examine the challenges posed by the evolution of resistance to synthetic gene drives and review the various design strategies adapted to reduce resistance. We also provide an overview of the recent efforts to develop mitigation measures to limit engineered gene drive spread or persistence either by reversing, neutralizing, or inactivating the drive components. Lastly, this review discusses the main applications of CRISPR-based gene drives across various species.

## 2. Overview of Different Types of Gene Drives

### 2.1. Natural Gene Drives

Naturally occurring gene drives (also known as meiotic drives) violate Mendel’s law of equal segregation and can rapidly spread through populations imposing in some cases an ecological fitness cost. The term meiotic drive was first announced in 1957 to refer to drive that occurs during gametogenesis [18]. These drives can control their transmission via various methods. For example, some drives work via transposable elements that overreplicate biasing transmission by increasing their numbers, whereas others work via accessory chromosomes, also called “B chromosomes,” that increase their transmission by moving towards germline cells and away from somatic cells [19, 20]. Besides those two examples, many other natural selfish genetic elements have been reported in the literature and this review will focus on the most documented ones.

Among the natural drive systems, the female meiotic drive (also known as a chromosomal drive) was encountered in several animal species, including Mus musculus [21, 22], Drosophila [23], and plant species such as Zea mays [24] and Mimulus (monkeyflowers) [25]. This natural phenomenon occurs when a selfish genetic element amends chromosome segregation during female meiosis to segregate to the egg and spread with high frequency to the next generation [26].

Male meiotic drive (also known as genic drive or sperm killers) is another characterized natural drive system that includes a driving element, which averts the maturation or function of sperm that do not contain it. The segregation distorter (SD) reported in the genus Drosophila [27, 28] and the t haplotype selfish genetic element identified in house mice (*Mus domesticus*) are the two most described examples [29]. In both examples, sperm that do not carry the drive element either degenerate as seen in Drosophila or become functionally impaired as observed in house mice [29].

First discovered in some populations of red flour beetle (*Tribolium castaneum*), MEDEA (maternal effect dominant embryonic arrest) are selfish genetic elements that cause embryonic lethality in individuals that did not inherit the elements from the female [30]. This selfish genetic element has later been adopted for the development of synthetic MEDEA systems in the fruit fly Drosophila and other invasive insect species to suppress pest populations as discussed later in this review.

Another type of drive exclusively found in the fungal kingdom is spore killers (Spok), which are selfish genetic elements that bias inheritance by interfering with sexual transmission [31]. Like all killer meiotic drivers, spore killers must differentiate between spores that inherit the drive locus and those that do not. Therefore, spores carrying the spore killer locus will survive, while those without the locus will be destroyed. Initially discovered in *Neurospora intermedia*, scientists reported that crosses between Spok+ and Spok− strains resulted in the death of half the progeny of a given mating. Recent work in the model fungus *Podospora anserina* has begun to unravel the mechanisms of spore killing [32]. This fungus contains multiple sets of Spok loci, but the most studied one is Spok1. Mating analysis between resistant and susceptible strains narrowed down SPOK1 to an individual protein surrounded by transposable elements. Once removed from the genome, spore killing ceases and segregation returns to normal. The exact mechanism of spore killing remains unknown as attempts to separate the two predicted domains of SPOK1 proved unsuccessful. SPOK proteins appear to be abundant across the fungal kingdom, including the plant pathogens *Nectria haematococca* (also known as *Fusarium solani*) and *Fusarium verticillioides*, the human pathogen *Aspergillus fumigatus*, and *Pseudogymnoascus destructans*, the causal agent of white-nose syndrome in bats [33, 34]. Additionally, Spok1 can be integrated into other fungi to generate drive in those organisms. As discussed later in this review, the discovery of the Spok1 coding region will pave the way for future work in gene drives against economically important fungal pathogens.

### 2.2. Synthetic Gene Drives

The use of natural drive elements to engineer synthetic gene drives and spread desired genes into populations was proposed as early as the 1960s as a method for pest control [35, 36]. This concept was brought to light again in 2003, with the potential use of specific enzymes named “homing endonucleases” [4]. Homing endonuclease genes (HEGs) are a class of selfish genetic elements able to copy themselves onto a homologous chromosome and encode components that mediate their own duplication. This duplication involves the introduction of a double-stranded break (DSB) on the homologous chromosome and repairing the DSB using the gene drive sequence as a template. Besides HEG, there are numerous types of selfish genetic elements used to engineer synthetic gene drives, many of them having unique modes of transmission and being elaborated further in this review. Advancements in genome engineering tools and mainly the integration of the widely used CRISPR-Cas9 system into gene drive designs have led to the establishment of highly effective genetic elements that efficiently promote their spread within a population via homology directed repair (HDR).

From the perspective of bioengineering efforts, synthetic gene drives provide a highly efficient platform for ensuring the inheritance of alleles, even when they might be detrimental to the organism’s fitness. They have widespread applications that fall into two categories: those engineered for population modification and those designed for population suppression. Modification gene drives can be engineered either to amend a trait in common pests and help saving a threatened species from fast-spreading diseases or to bring in new traits to a population and pass it down to the progeny. On the other hand, population suppression systems are projected to eradicate completely a nonindigenous invasive species, a disease vector, or a crop pest [37]. Here, we discuss the main synthetic drive systems engineered to date.

#### 2.2.1. Non-CRISPR Synthetic Gene Drives

*(1) Homing Endonuclease-Based Synthetic Gene Drives*. Before the establishment of the CRISPR-mediated adaptive immune system and its associated proteins (Cas), transcription activator-like effector nucleases (TALENs) and zinc-finger nucleases (ZFNs) were used as platforms for engineering homing-based gene drive systems in *Drosophila melanogaster* [38]. Data from this study provide proof of principle that both TALEN and ZFN nucleases can be used to create synthetic selfish elements (SSEs) that can spread into the genome of naive Drosophila populations. The overall transmission rates recorded for these synthetic SSEs were higher than those observed with the natural homing endonuclease (I-SceI) [39]. However, the usage of these nucleases in gene drive research was hampered shortly after this first attempt due to their limited stability and tendency to recombine during homing [38].

*(2) Other Synthetic Gene Drives*.

*Toxin-Antidote-Based Gene Drives*. Toxin-antidotes (TAs) are selfish genetic elements that spread in populations by selectively killing noncarrier individuals. Several forms of TA gene drives have been studied so far. Initial forms of TA systems work by chromosomal rearrangements, and these drives have been engineered in various species of mosquitoes and flies including Anopheles and Drosophila as described in a separate section as follows. Another way for TA systems to work is by using an RNAi lethal toxin along with a zygotically expressed rescue element or antidote. The maternally expressed toxins used to date have been microRNAs (miRNAs) targeting essential genes [40]. This system can be designed in two different ways. When the expressed toxin and the zygotically expressed antidote are introduced on two closed loci on the same chromosome, it is called MEDEA. However, if the design consists of two constructs on different chromosomes, each carrying a maternally expressed toxin and a zygotically expressed antidote that suppresses the activity of the toxin, it is known as underdominance (UD^MEL^) [41]. Several synthetic RNAi-based TA gene drives have been developed theoretically or tested on mosquitos and flies to control diseases spread by those vectors. Some of these developed drives require a significant introduction threshold to ensure their spread, which makes them suitable to small secluded area populations with reduced gene flow [42–47]. The main RNAi-based TA drive systems developed to date are summarized in Table 1. Operating similarly to RNAi-based drives, some established TA systems are CRISPR based. The advantage of CRISPR-TA gene drives over others is their high flexibility and ability to be implemented in diverse species [48]. In these systems, the “toxin” element is the Cas9 along with gRNAs designed to disrupt an essential gene on the WT chromosome. The “antidote” element is a functional copy of the target gene positioned within the drive allele and recoded in a way to not match the gRNAs to escape the disruption by the drive. Therefore, individuals who inherit both the toxin-disrupted allele and the antidote will be protected from the lethal toxic effect of the drive [48]. The CRISPR-TA drive systems developed to date will be discussed in subsequent sections.

**Table 1:** RNAi-based toxin-antidote gene drive systems developed.

Drive type	Species/organism	Design	Application	Reference
MEDEA	Drosophila	Transposable element vector in which the maternal germline-specific bicoid promoter drives the expression of a polycistronic transcript encoding two microRNAs (miRNAs) designed to silence expression of the gene producing the toxin, myd88	Population replacement	[49]
MEDEA	*D. suzukii*	Maternal miRNA “toxin” targeting the essential gene myd88 linked to an “antidote” consisting of myd88 coding region driven by the embryo-specific bnk promoter	Population replacement	[46]
MEDEA	*Drosophila melanogaster*	The experimental design consisted of two new synthetic MEDEA elements based around the two genes dah and o-fut1, involved in cellular blastoderm formation or Notch signaling	Population replacement	[44]
	Theoretical design on *D. melanogaster*	The theoretical design relies on environmental cues that could be used to cause a population crash. The MEDEA will be given time to spread before the cue appears and result in cue-dependent suppression	Population suppression	
Inverse MEDEA	Theoretical design (not tested experimentally on any species)	The designed system consisted of 2 genetic components: a zygotic toxin and maternal antidote that result in heterozygous progeny of wild-type (WT) mothers being unviable	Population replacement	[50]
UD^MEL^	*D. melanogaster*	The designed system consists of a construct harboring an RNAi knockdown dsRNA targeting an haploinsufficient gene RpL14 and an RNAi-insensitive RpL14 rescue	Population replacement	[43]
UD^MEL^	*D. melanogaster*	UD^MEL^ system consists of 2 constructs: UD^MEL^-1 comprises maternal toxin A and zygotic antidote B, and UD^MEL^-2 includes maternal toxin B and zygotic antidote A. Both toxins target essential genes for normal embryonic development	Population replacement	[42]
UD^MEL^	Theoretical design on *Aedes aegypti*	UD^MEL^ system comprises two unlinked constructs. The maternally expressed toxin is designed on the first construct, whereas its zygotically expressed antidote is present on a separate construct	Population replacement	[47]
Combined MEDEA-underdominance system	Theoretical design (not tested experimentally on any species)	The system combines MEDEA and underdominance designs in one construct on a somatic chromosome	Population replacement	[51]
Medusa (sex chromosome-associated MEDEA underdominance)	Theoretical design on *A. gambiae*	Medusa system consisted of four components: a maternally expressed X-linked toxin and a zygotically expressed Y-linked antidote, a zygotically expressed Y-linked toxin, and a zygotically expressed X-linked antidote	Population suppression	[52]

*Engineered Reciprocal Chromosome Translocation Drive*. Reciprocal chromosome translocations result in the mutual exchange of the DNA between terminal segments of two nonhomologous chromosomes. This gene drive was initially proposed in 1940 and 1968 as an approach to attain high-threshold pest population replacement [53, 54]. Few years later, a first field experiment using chromosome translocation was conducted on mosquitoes [55]. Afterwards, a similar drive system was established in the spider mite *Tetranychus urticae*, a haplodiploid species in which there is a substantial selection in haploid males for fit homozygotes [56]. Recently, Buchman et al. [57] reported the generation of engineered translocation-bearing strains of *D. melanogaster* through targeted chromosomal breakage, using a site-specific nuclease, followed by homologous recombination. Findings from this study revealed high threshold population replacement in laboratory populations, while it remains to be explored in wild populations. Likewise, a recent modeling study supported the use of translocations for spreading disease-resistant genes into *Aedes aegypti* populations in a confined and reversible way [47].

*Spore Killer Synthetic Gene Drives*. This system is one of the best-studied natural drive systems explored in various fungal species such as Neurospora, *Podospora anserina*, and *Schizosaccharomyces pombe* [58–62]. This drive system acts by protecting the gametes that inherit the drive element and killing those that did not inherit it. Inspired by this natural drive system, a synthetic spore killer drive system has recently been developed on the cereal pathogen *Fusarium graminearum*, responsible for head blight (FHB) disease on multiple hosts [63]. In this paper, the authors demonstrate that the natural gene drive element Spok1 can effectively alter the inheritance of two virulence-associated loci in *F. graminearum*. The first-targeted locus comprises the trichothecene gene cluster while the second is the ABC1 transporter gene shown to be implicated in virulence and tolerance to xenobiotics. Findings from this study opened new research avenues and raised the question of the feasibility of using synthetic gene drives in the future as a control strategy to mitigate fungal disease in plants.

*Synthetic Meiotic Drive Systems*. Synthetic sex-ratio distorter (SD) is a drive system developed to control heterogametic pest species by hampering the viability and development of one of the two heterogametes during germline formation and triggering a bias in the reproductive sex ratio [41]. The malaria vector, *A. gambiae*, is the first organism in which a synthetic SD system was successfully established. This system was engineered by expressing the I-PpoI endonuclease targeting ribosomal DNA repeats that occur exclusively on the X chromosome [6]. Later, this same group investigated the mechanisms regulating SD in this species [64]. Findings from this study have shown that the observed male bias is potentially due to sperm carrying a damaged sex chromosome which reduce their ability to fertilize the female oocyte rather than loss of fertility in endonuclease cleaved heterosomes. Simoni et al. were able to combine this sex distorter with a gene drive and validate a successful sex distorting gene drive (SDGD) [65]. Another synthetic meiotic gene drive construct named t-Sry has been theoretically proposed in various studies as a measure to exterminate the invasive population of house mice [66, 67]. This construct comprises the naturally occurring autosomal meiotic driver (t haplotype) and the male determining Sry gene, localized on the Y chromosome. As described earlier, the t haplotype is a sperm killer that damages non-t-bearing WT sperm, resulting of its inheritance by more than 90% of the progeny of t-carrying males. Therefore, to guarantee the spread of the Sry gene to both XX and XY offspring, this gene must be copied and inserted into the t haplotype. This ensures that all offspring receiving the t-allele with the Sry gene would be genotypically XX t-Sry, but phenotypically sterile male due to the lack of Y-linked genes crucial for sperm development [66, 67].

#### 2.2.2. CRISPR-Based Synthetic Gene Drives

**Figure 1:**
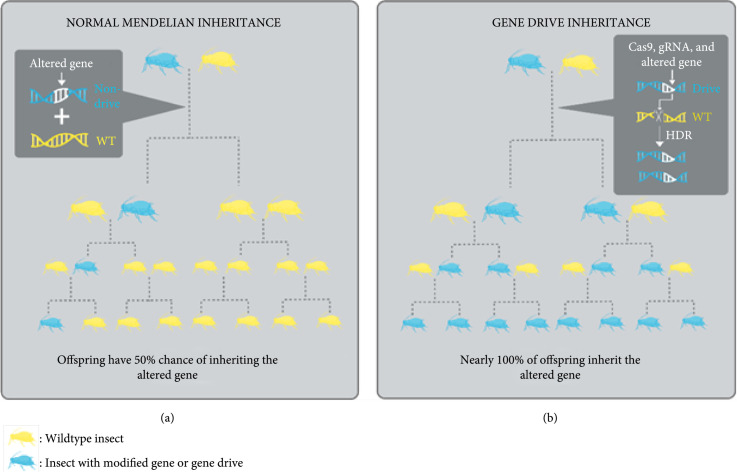
Illustration of CRISPR-based gene drive inheritance compared to natural Mendelian inheritance. Insects and other organisms typically have a 50% chance of passing an altered gene to their progeny (a). When inherited this way, the altered gene may not spread to many individuals in the population and will vanish after many generations. However, if the altered allele is linked to a CRISPR/Cas9 gene drive as shown in the schematic on the upper-right side of (b), the CRISPR gRNA will guide the Cas9 to the target site on the WT chromosome to make a double-strand break at the target site. The break will be repaired through HDR using the drive-containing chromosome as a template. This will result in the gRNA, the Cas9 drive, and the altered gene being copied into the homologous chromosome guaranteeing that it gets passed at higher rates (b).

Clustered regularly interspaced short palindromic repeat- (CRISPR-) mediated genome editing is a powerful genetic engineering tool that is rapidly developing. The canonical CRISPR ribonuclease *Streptococcus pyogenes* Cas9 has been adopted for applications beyond gene editing, such as a transcriptional regulator and epigenetic and chromatin modifiers [68]. One of these applications is its use in gene drives for generating super-Mendelian inheritance of target genes in insects and mice [69, 70]. Figure 1 depicts a schematic representation of the natural Mendelian inheritance in comparison with the forced inheritance using CRISPR-based gene drives.

Maintaining and spreading a desirable mutation can be achieved by taking advantage of the versatility of CRISPR/Cas9 genome engineering tools. The principal method of CRISPR/Cas9 gene drives involves the introduction of a segment of DNA that carries a passenger sequence to be maintained along with a Cas9/gRNA coding sequence that allows its introduction into a target locus via double-stranded break repair mechanism resulting in its duplication. The various applications of this tool will be discussed in detail in a subsequent section.

## 3. Resistance to Synthetic Gene Drive Systems and Preventive Approaches

A key challenge in the design and use of gene drives has been the development of resistance, averting their spread. Resistance to natural drive systems has remained poorly understood [45]. On the contrary, synthetic gene drive systems offer some of the most enlightening studies on the evolution of resistance. In these synthetic systems, the resistance evolution is mainly a result of mutations at target genomic loci resulting in the suppression of drive machinery, as witnessed in many drives, including homing-based gene drives [69, 71–73] and MEDEA and underdominance-like systems [46].

The major efforts for evading resistance have been applied in CRISPR gene drives as described here. However, the prevention of resistance to synthetic gene drive elements cannot be fully attained but can be minimized by the vigilant drive design. In their effort to better understand and combat drive resistance in homing-based population suppression systems, Marshall et al. [74] have established a mathematical model to estimate the maximum acceptable resistant allele generation rates to attain stable and long-term suppression of various populations. Findings from this study revealed an inverse correlation between the rate of resistant allele generation and the number of multiplexed gRNAs. Along the same line, Champer et al. [75] have explored different strategies for reducing resistance to CRISPR homing gene drives in this same species. The use of multiple gRNAs targeting neighboring sites in the target gene was proven to be efficient at improving drive efficiency and reducing resistance rates, particularly in the germline. In this same study, authors presented an autosomal drive able to successfully achieve conversion in the male germline without developing resistance alleles in embryos through paternally transferred Cas9. Lastly, the use of different promoters for Cas9 expression was another effective approach to modulate resistance mechanisms and rates, with the nanos promoter being a better option to evade high levels of resistance compared to the vasa promoter. A subsequent study by this same group [76] evaluated the performance and efficiency of homing drives using multiple gRNAs in both *D. melanogaster*, using modeling to apply conclusions to drives in Anopheles mosquitoes. Findings from this work revealed that each type of drive has an optimal number of gRNAs to achieve the maximal performance. These results controvert the old belief that drive efficiency can be indefinitely improved by adding more gRNAs and emphasize on the necessity of combining approaches to hamper resistance evolution in CRISPR homing drives.

Recently, we witnessed the engineering of three homing split drives carrying a recoded rescue copy of the target gene as a measure to overcome resistance. In the study by Kandul et al. [77], they engineered a “home-and-rescue” split-drive system in insects following specific design criteria. These include targeting the 3′ coding sequence of a vital gene essential for insect viability, encoding a dominant rescue of the endogenous target gene into the home-and-rescue drive, and using exogenous 3′untranslated region (3′UTR) to prevent expected deleterious recombination events between the drive and the endogenous target gene. This system was tested in Drosophila to reduce the accumulation of drive-resistant alleles significantly. Similar gene split drive systems targeting essential genes required for viability and fertility were developed in the study by Terradas et al. [78]. Prior to those two studies, Champer et al. [79] have used this same tactic of providing a rescue allele along with the homing drive but the design included two gRNAs that target a haplolethal gene in Drosophila. This led to a significant reduction of both functional and nonfunctional resistance alleles [79], further emphasizing on the advantages of combining multiple safeguards to reduce resistance to gene drive spread.

Recently, mathematical and computational models were also developed to find conditions under which drives could evade resistance [80]. In their recent computational modeling study, Faber et al. [81] combined a homing gene drive with a cleave and rescue (ClvR) gene drive in *Sciurus carolinensis*, an invasive pest that caused serious economic losses in the United Kingdom. The ClvR drive works by directing Cas-gRNA(s) to induce breaks in an essential gene and providing a recoded rescue version of this gene in the drive cassette. Therefore, only the descendants inheriting the rescue will survive. Findings from this study show that the HD-ClvR drive could potentially eradicate resistance alleles that usually form during gene drive homing and localize spread. To conclude, it is worth mentioning that regardless of the approach adopted and its promising effects on improving resistance during laboratory experiments, it might still be unsuccessful once released in the wild for various unknown reasons.

## 4. Containment Strategies for Synthetic Gene Drive Organisms

Various ecological, physical, and molecular approaches have been proposed to limit or reverse changes caused by released synthetic drives and hamper their spread [82]. Performing experiments outside the habitat of the drive organism or in areas without potential wild mates, the use of physical barriers to separate the drive organisms from the environment, and the use of experimental strains unable to reproduce with wild organisms were the most straightforward proposed confinement approaches [83].

Among the molecular concepts anticipated to restrict the uncontrolled spread of synthetic gene drive were as follows:

(i) A “reversal” drive that can be released subsequently to overwrite genomic changes spread by a first drive that was released without consent or that produced unexpected side effects [13]
(ii) An “immunizing” drive that can be used to render populations immune to a specific unwanted drive by recoding sequences targeted by that drive [13]
(iii) “Split” drive, a so-called safeguarding drive, for which the components of the drive have separate genomic loci in such a way that only a certain part of the information for a functionally active drive is inherited [9, 82]
(iv) A “daisy” drive that consists of a linear series of unlinked drive elements located on separate chromosomes. In this hypothetical system, the first drive element is responsible for duplicating the second and the second for duplicating the third, etc. Yet, elements at the bottom of the chain cannot drive; thus, the whole drive system gets sequentially lost over time [84]
(v) A “self-eliminating” gene drive that consists of the gene drive transgene, a marker gene, and cargo genes along with a gene that encodes a recombinase. For this design, the entire cassette should be flanked by analogous recombination sites. Therefore, when the recombinase is expressed, the two flanking regions will recombine to excise the gene drive construct and restore the host allele [85]. Other potential designs for self-eliminating CRISPR/Cas9-based gene drive were proposed in this study, including an integration-deficient transposase delimited with a corresponding inverted terminal repeat. The last proposed design includes a direct repeat (corresponding to the WT host allele) that flanks the gene drive and related transgenes. This design can result in the loss of all transgene sequences via a DNA break repair identified as single-strand annealing [85]

Of the proposed molecular containment strategies, only a few have been tested experimentally, while the proof of efficacy of others is yet to be established. DiCarlo et al. [9] validated the first CRISPR-Cas9 split-drive strategy in *S. cerevisiae*, for which Cas9 was encoded on a separate plasmid from the drive. However, Cas9 cannot be encoded in wild yeast populations; thus, the spread of the drive is not possible in these organisms. In this same study, the authors have tested another easy-to-use confinement method and showed over 99% efficiency for a reversible drive to overwrite an earlier drive [9]. Similar safeguarding gene drive systems were later engineered in the fruit fly *D. melanogaster* [86] and the human disease vector, *A. aegypti* [87]. In recent years, we also witnessed the design and assessment of new self-propagating genetic-neutralizing elements in *D. melanogaster* that can either disable Cas9 carried by a gene drive (e-CHACR (erasing construct hitchhiking on the autocatalytic chain reaction)) or remove and substitute the gene drive (ERACR (element reversing the autocatalytic chain reaction)) [88]. Both designed elements encode only gRNAs without a Cas9. The e-CHACRs can be randomly inserted into the genome and encode multiple gRNAs, one of which will cut at the genomic insertion site of e-CHACR, to enable self-replication in the presence of Cas9. The additional gRNA(s) are designed to target, cleave, and inactivate the Cas9 component of the gene drive. On the contrary, ERACRs are usually inserted at the same genomic locus as the gene drive and encode for two gRNAs that will interact and form a complex with the Cas9 to generate cuts on both sides of the drive element to remove it and substitute it [88]. Data from this study revealed that e-CHACRs were efficiently able to inactivate Cas9 but with diverse transmission frequencies, whereas ERACRs were able to frequently delete and replace a gene-drive element.

Another novel genetic approach has recently been engineered and tested in the malaria vector *Anopheles gambiae*. This approach involves expressing an anti-CRISPR protein (AcrIIA4), able to inactivate CRISPR-based gene drives and reinstate the Mendelian rates of inheritance. Data from this study showed that a single release of male mosquitoes carrying the AcrIIA4 protein was able to cease the spread of a highly active gene drive, averting the population crash [89].

Besides the above-discussed strategies, one of the most propitious measures for drive confinement has been the introduction of threshold-dependent gene drives that can only spread when present at frequencies above critical thresholds compared to the WT counterparts. However, when existing below this frequency, the drive allele will be lost from the population. These drive systems have numerous benefits making them well suited for contained population modification, including high invasion thresholds, reduced chance of escape, and ability of reversal or “drive out” through releases of WT organisms. Over the years, a number of threshold-dependent gene drive systems have been proposed and developed, to cite a few [47, 90–92].

Along the same line, a novel approach, the “locally fixed alleles,” was proposed as a method to localize a gene drive to small island populations that display a significant degree of genetic isolation from neighboring populations [93]. This approach consists of a CRISPR/Cas9 drive targeting locally fixed alleles in this small population and can spread to individuals carrying these alleles. Individuals that do not carry these specific alleles will be naturally resistant to the drive.

## 5. Application of CRISPR-Based Gene Drives in Various Organisms

### 5.1. Applications in Animals

#### 5.1.1. Mosquitoes

Mosquitos have been a target for gene drive development due to their significance as vectors for various human diseases. Many studies have developed methods to ensure the efficient spread of transgenes within wild mosquito populations, leading to attenuated capacity to transmit disease or wholesale population decrease. Mosquitos with genome modifications that introduce female-specific lethal genes into a WT population via transgenic males have been developed to control populations of *Ae. aegypti* [94] and *An. stephensi* [95]. Release of CRISPR-mutated sterile males [96] or gene knockouts that result in flightless females [97] have also been developed to suppress female fertility in *Ae. aegypti*. These methods rely on introducing transgenes via the continued release of engineered individuals. The use of gene drives is an attractive strategy for achieving high transgene penetrance with reduced transgenic animal introductions [98].

An efficient gene drive that may be useful in delivering antiparasite effectors has also been demonstrated in *An. gambiae* [99, 100]. This gene drive is integrated into an ommochrome biosynthetic gene (Agcd) and carries fluorescent and morphological markers that are useful in tracking the spread of the gene drive. Outcrossing of first-generation gene drive lines with the wild type led to >95% of drive propagation in progeny, and this efficiency remained high to give an average of 96.7% over four generations. The low fitness penalty associated with this gene drive, either hetero or homozygote, also makes it an attractive platform for delivering desired cargo genes into a population.

*(1) Gene Drives for Gene Replacement*. *Anopheles stephensi* is a malarial vector in the Indian subcontinent that presents an attractive target for the development of a disease-combating gene drive. Gantz et al. achieved high gene conversion rates with a CRISPR-based gene drive [69]. They tested a ~17 kb gene drive carrying Cas9, driven under a germline-specific promoter (male and female), and demonstrated gene conversion in 99.5% of progeny from outcrossing of transgenic lines. Both genders derived from transgenic female mothers produce progeny with a high frequency of resistance mutations in the targeted genome sequence (due to nonhomologous end joining (NHEJ) repair), resulting in near-Mendelian inheritance ratios of the gene drive transgene [69]. Eggs, which are expected to have drive component molecules, permit the formation of active Cas9 nuclease. This observation demonstrates that a functioning host HDR machinery is critical for the success of a CRISPR gene drive. This gene drive was also capable of delivering antipathogen effector genes as cargo. These genes were transcriptionally activated upon blood feeding, demonstrating the expected transcriptional response of blood meal-inducible promoters and utility of this gene drive in combating disease transition.

*(2) Gene Drives for Population Control*. Reduction of mosquito populations is one strategy for combating disease spread, and CRISPR gene drives that have either lower fertility [99, 101] or distort sex distribution within a population [65] have been developed to achieve this goal.

Hammond et al. developed a gene drive able to reduce female fertility and spread within a caged population of *An. gambiae* [101]. This gene drive was targeted to disrupt haplosufficient fertility genes identified by gene expression correlation for the likelihood of female sterility in *D. melanogaster* and ovary-specific expression. Homozygotic mutant females at these three genes were sterile, whereas heterozygotes showed normal fecundity when loci were disrupted by a GFP-expressing construct. Gene drives targeting each gene resulted in super-Mendelian inheritance at the target loci when heterozygotic individuals of either sex were crossed with the wild type at gene conversation rates between 87 and 99.3%. The effect on fertility was even more dramatic, where no embryos were recovered from a heterozygotic female carrying a driver construct at AGAP005958. The highest fertility observed was 9.3% (AGAP007280) compared to that of the wild type. Despite the high gene conversion rates observed, reduction in heterozygotic fertility in gene drive-converted females will limit the utility of the drive in wild populations.

Only *An. gambiae* female insects transmit diseases, and biasing the sex distribution in a population is an attractive approach. The CRISPR gene drive was used to disrupt the critical sex determinant doublesex (dsx) gene in *An. gambiae* leading to intersex insects. Disruption of the intron 4/exon 5 boundary prevents the formation of an exon 5-containing, female-specific, splice variant without affecting the male-specific transcript. Female mosquitos that were homozygous for the disrupted allele were sterile and displayed intersex reproductive organs whereas males develop normally. The gene drive was able to drive homing in >95% of progeny of both male and female heterozygotic parents with minimal reduction of larval output of heterozygotic females. In cage studies, the driver allele frequency reached 100% within 12 generations from a starting frequency of 12.5% (50% of males were heterozygotic for the gene drive in an initial population of 50% male and 50% female). Minimal indels at the site of the gRNA target were observed (1.16%) which would have led to gene drive resistance [99].

CRISPR-based genetic tools that remove the X sex chromosome during spermatogenesis and bias the sex makeup of progeny have been developed [65]. Simoni et al. developed a sex-distorter gene drive (SDGD) that leads to a super-Mendelian inheritance of the X chromosome-shredding I-Ppol nuclease, coexpressed with a CRISPR-based gene drive inserted into the conserved doublesex (dsx) gene. The population of sperm from a gene-drive carrier will be highly biased towards Y sex chromosome bearing, leading to a more significant proportion of the progeny being male. This SDGD led to all-male populations within 10–14 generations from a starting allelic frequency of 2.5% and equal sex makeup. I-Ppol (which cleaves the conserved X-linked ribosomal gene cluster) was autosome expressed because meiotic sex-chromosome inactivation would preclude its transcription from sex chromosomes during gametogenesis. The beta2 promoter for I-PpoI also had to be modified to reduce the expression to prevent sterility associated with high levels of I-PpoI.

#### 5.1.2. Drosophila

The maintenance of a gene drive in a target population is counteracted by multiple resistance mechanisms, including the rise of gene drive-resistant mutations at the homing locus. These mutations, termed r1 (maintain a functional gene) and r2 (lead to loss of function), pose different challenges [75]. To overcome these challenges, toxin-antidote CRISPR gene drives that contain a recoded gRNA-resistant version of a target gene alongside a gRNA targeting the native copy have been developed in flies [48, 102].

In the cleave and rescue (ClvR) system developed by Oberhofer et al., a toxin-antidote gene drive, introduced on an autosomal chromosome, was designed to target an X chromosomal essential gene [102]. The inheritance patterns create conditions in which ClvR-bearing parents transmit a potential fitness cost to WT chromosome-bearing progeny due to gene conversion and maternally transmitted Cas9 activity in the zygote. These conditions promote a relative increase in the frequency of ClvR-bearing chromosomes. The success of this gene drive was assessed in cases where the targeted essential gene is haplosufficient (ClvR^tko^). ClvR^tko^ led to the inactivation of >99% of tko genes with minimal observed functional resistance (r1) mutations. Similar to ClvR, Champer et al. developed a “same-site” toxin-antidote drive such that the drive and target loci are on the same recessive lethal gene [103]. This approach brings the advantages of ClvR while potentially improving the efficacy of rescue by placing the antidote under its native promoter.

Another effective TA split gene drive with two guide RNAs targeting adjacent sites of a haplolethal gene (RpL35A, a conserved protein component of the 60S ribosomal subunit) and a gRNA-resistant rescue allele for the essential gene has been tested [79]. The frequency of drive carriers rose from 32% (present as homozygotes) in generation 0 to 100% in generation 6 when eggs from the gene drive and Cas9 homozygotes were mixed with eggs from Cas9 homozygotes. The design utilized here allows for efficient drive homing and fixation in the presence of Cas9 expressed from a separate locus. The success of this strategy is reliant on the stringent selective pressure imposed by gene copy loss and use of multiple gRNAs that improve drive homing, which led to >90% inheritance in the offspring resulting from the cross between heterozygous parents and wild type. Moreover, the progeny of gene drive heterozygous females and WT males led to reduced egg-to-pupa survival rates compared to heterozygotic male parents (55 vs 80%), indicating r2 (nonfunctional) allele formation due to maternal deposition of targeting Cas9. A substantial number of embryos received mutated alleles that were later undetectable in adults. This indicates that a portion of the r2 alleles was eliminated during embryonic development.

A split-drive design developed by Kandul et al. [104] was capable of targeting multiple host genes. This gRNA-mediated effector (GME) drive, where two gRNAs are expressed from a homing cassette (one for homing locus on the X chromosome and 2^nd^ gRNA for the mutation target (yellow locus)) and Cas9 from a separate locus, accomplished both goals. Cas9 was expressed from an integrated locus on chromosome III and driven from one of 4 promoters; nanos (nos) and vasa (vas) promoters to drive expression in early germline Bicaudal C (BicC) in late germ cells, and ubiquitin 63E (Ubi) in somatic cells. Homo- or hemizygotic gRNA lines crossed with heterozygotic Cas9 lines resulted in both gRNA targets being modified to varying degrees based on the gender of the parents. Maternally deposited Cas9 leads to gene conversion in F1 transheterozygous female progeny for all Cas9 promoter lines. Progeny of Cas9 males generally showed reduced gene conversion. Both genes were targeted in all-female progeny when Cas9 is driven by nos and vas, Ubi driven Cas9 led to 100% white and 91.3% yellow LOF, and BicC led to white in 64.3% but no yellow mutations. Indel formation was higher (26 or 27%) for maternally deposited Cas9 under the Ubi promoter than paternally expressed Cas9 (9.9%) in mitotic oocytes due to lower HDR repair efficiency. A significant decrease in the homing rate between F2 progeny and F3 progeny was observed for all Cas9 promoter-driven expression but not in LOF mutations at the homing and target genes. Accumulation of indels at these nonessential genes that preclude cleavage by the homing gRNA is a predicted outcome that leads to the emergence of resistant alleles. Overcoming these limitations will be essential for realizing the utility of this GME drive.

#### 5.1.3. Rodents

Although gene drives have been effectively used in insects, the development of mammalian drives is in the early stages [70, 105, 106]. An approach that demonstrated the first successful mammalian gene drive utilized a split drive design and achieved up to 72% gene conversation rates [70]. The gene drive was made up of a gRNA-bearing construct, Tyr^CopyCat^, targeting the tyrosinase (Tyr) gene integrated at the Tyr locus. Tyr^CopyCat^ lines were crossed with “constitutive” Cas9 transgenic lines to introduce Cas9. Ubiquitously expressed Cas9 construct led to the conversion of the receiver allele to null via indel introduction, but not HDR and subsequent copying of Tyr^CopyCat^. A more successful strategy was to conditionally drive Cas9 expression by utilizing Cre recombinase, removing a loxP flanked stop codon, and allowing expression of Cas9 in the meiotic germline. This strategy improved the likelihood of HDR being used to repair CRISPR-mediated DSB over NHEJ and an increased gene conversion rate. The approach was more effective during oogenesis over spermatogenesis, suggesting that the timing of Cas9 expression better coincided with meiotic chromosome pairing and active meiotic recombination machinery in maturing eggs.

Attempts to utilize gene drives with a direct Cas9 expression construct have been less successful. Introducing CRISPR-mediated DSB during gamete maturation that coincides with an active HDR machinery to driver allele conversion has proven challenging. Expression of Cas9 under the direct control of either zygotic or germline promoters led to the accumulation of indels at the gRNA target site, indicating functional Cas9-gRNA complex formation, but no gene conversion was observed [105]. Pfitzner et al. drove Cas9 expression in the early embryo under the ubiquitous CAG promoter, as well as the germline Vasa promoter, and found that this strategy only succeeded in introducing indels at the target site. A new gene drive that introduced Cas9 into the endogenous Spo11 locus was developed to improve the timing of Cas9 expression [106]. Spo11 is required for DSB formation in the prophase of meiosis I to allow meiotic recombination. Cas9 was expressed as a fusion with SPO11, separated with a self-cleaving peptide. This strategy allowed drive homing in both sperm and eggs. However, low rates of absolute gene conversion were observed, likely due to the low levels of Cas9 expression.

These results present an opportunity to identify alternative strategies that can drive Cas9 expression in the meiotic stage of germline maturation to allow for efficient gene drive homing. Although demonstrably successful and promising, variable gene conversion rates in mice will require optimization [107].

### 5.2. Applications in Microorganisms

The first microbial CRISPR gene drive system was developed and optimized in the budding yeast *Saccharomyces cerevisiae* [9]. Since then, multiple studies have reported the validation of CRISPR-Cas9 RNA-guided gene drive systems for use in this organism and assessed various modes of control and regulation of gene drives in vivo. In their first study, DiCarlo and colleagues constructed multiple gene drives to examine various hypotheses [9]. They first tested the ability of synthetic gene drives to convert the ADE2 gene that encodes a phosphoribosylaminoimidazole carboxylase to a nonfunctional copy of this gene (ade2). The success of this approach was evaluated on laboratory yeast strains as well as WT populations. By mating haploid yeast containing either the mutated or the WT gene, the resulting diploids inherited both copies. However, the ade2 variant was carried out by more than 99% of their following haploid offspring (rather than the expected results of 50% of the offspring carrying each variant). This indicates an effective overwriting of the WT genotype (ADE2). The success of this tool was validated on another essential gene, ABD1. This first study sets the foundation for future efforts to design and implement effective and steady gene drives designated for real-life practical applications [9]. A few years later, Yan and Finnigan [108] optimized a multilocus CRISPR gene drive system in this same organism that consists of a single copy of *S. pyogenes* Cas9 and three guide RNAs targeting the loci HIS3, SHS1, and DNL4, to propagate three gene drives.

Subsequently, more studies have been conducted in *S. cerevisiae* to establish approaches to control, regulate, or inhibit drive systems in vivo. In this regard, Roggenkamp et al. [109] reported the drive activity to be contingent on four conserved features: the Cas9 protein level, the sgRNA uniqueness, the Cas9 nucleocytoplasmic shuttling, and lastly, the novel Cas9-Cas9 tandem fusions. In their latest study, Goeckel et al. [110] expanded upon their previous work to assess the ability to modulate the nuclease activity of Cas9 in *S. cerevisiae* based on its nucleocytoplasmic localization. Authors examined nonnative nuclear localization sequences (both nuclear localization signal (NLS) and nuclear export signal (NES)) on Cas9 fusion proteins and demonstrated that mutational substitutions to nuclear signals and combinatorial fusions could modulate the gene drive activity within a cell population [110].

More recently, Xu et al. [111] established a strategy of meiotic drive, labeled “chromosome drive” in *S. cerevisiae*, in which they utilize chromosomes as drive cassettes to achieve biased inheritance of large sections of DNA. Data from this study validate that using CRISPR-Cas9, a whole chromosome could be removed via a single DSB that occurs close to the centromere.

After their work on *S. cerevisiae*, Shapiro and colleagues developed a CRISPR-Cas9-based “gene drive array” (GDA) platform to facilitate the tracking of genetic manipulation in the pathogenic yeast *Candida albicans* [112]. They first optimized the gene-drive deletion system by targeting the ade2 gene as its deletion yields a visually quantifiable phenotype. Unlike their previous findings in *S. cerevisiae* where one gRNA was enough to ensure effective replacement of alleles, it proved more challenging to design one gRNA to efficiently induce driving in *C. albicans*. However, a significant increase (~86-98%) in drive efficacy was observed when two gRNAs are designed on the 5′and 3′ ends of the target gene near the regions of donor homology. Upon confirming this technology, the authors scaled up the approach and created drives targeting genes encoding efflux pumps responsible for developing antifungal resistance and adhesins implicated in biofilm formation.

It was not until 2019 that the first bacterial CRISPR-based gene drive system was developed in the most common foodborne pathogenic bacterium, *Escherichia coli* (*E. coli*) to overcome antibiotic resistance. In this study, Valderrama et al. [113] validate a self-amplifying proactive genetic (Pro-AG) system that efficiently copies a functional gRNA cassette targeting the beta-lactamase (bla) gene associated with ampicillin resistance in *E. coli*. This study provides the first evidence of a selfish synthetic gene element propagation in bacteria. However, this design requires the presence of accessory constructs to ensure spread through a clonal population. Therefore, additional development is needed to create a self-contained bacterial gene drive.

Previously believed to be restricted to organisms that reproduce sexually, a recent pioneering study by Walter and Verdin [114] disproves this theory by reporting the success of the first CRISPR-Cas gene drive system developed in DNA viruses. This gene drive strategy developed in herpesvirus does not rely on sexual reproduction but on the ability of this virus to undergo a high level of homologous recombination during its replication cycle. When a given cell is coinfected by a wild-type and an engineered virus, this will generate a cut and replacement by HDR resulting in gene drive viruses that would invade the WT population. The viral gene targeted in this study was UL23, and the system’s efficiency was confirmed in cell culture experiments [114]. It will be interesting to test in the future if similar drive systems can be engineered to target other viruses and how this tool can be improved for use in animal models and eventually on human patients.

To date, we have not witnessed the development of any synthetic CRISPR-based gene drive system in the most intractable microbial group, filamentous fungi.

### 5.3. Applications in Plant Systems

The application of CRISPR gene drives in plants is still in its infancy due to NHEJ preference over HDR for double-stranded DNA break repair. In a groundbreaking publication, Zhang et al. reported the first efficient gene drive in plants for generating homozygotes in F1 progeny of *Arabidopsis thaliana*, surpassing the limitations of Mendelian genetic inheritance [10].

The autonomous gene drive that Zhang et al. [10] developed carries Cas9, two guide RNAs (gRNA) targeting the CRY1 gene or the ends of the gene drive cassette for release from the source plasmid, two fluorescent markers, and homology arms complementary to the insertion target locus. Authors compared the efficiency of driving Cas9 under multiple promoters and found that expression of Cas9 under the hybrid egg cell-specific promoter DD45/EC1.2 led to the highest efficiency in introducing the gene drive into T0 plants (4.68%). To test zygotic conversion by the gene drive, homozygous T2 Col ecotypes were crossed to polymorphic Ler WT plants and 5 out of 195 progenies were found to be true F1 homozygotes. Additionally, the authors found that the region surrounding the insertion site on the CRY1 locus was from the nondriver expressing ecotype demonstrating a highly defined HDR junction during gene conversion. Self-pollination of heterozygotic F1 plants led to a significant increase in the proportion of gene drive homozygotes over the expected Mendelian inheritance (37.2% observed vs 25% expected, p $-1.2\times 10^{-6}$, one-sample binomial test) and a corresponding decrease in the proportion of heterozygotes. These observations were considered strong evidence that the gene drive effectively propagates in a heterozygotic plant. However, the method of quantifying drive conversion in this study could be affected by the formation of resistance alleles, so it remains unclear if any drive conversion occurred.

To our knowledge, this was the first attempt to develop a gene drive in plants yet additional work is needed to improve the efficiency of the system. As mentioned earlier, double-stranded breaks repaired by NHEJ are the main obstacle to effective gene drives in plants. This also increases the development of resistant alleles making it challenging to accurately detect WT alleles and estimate allele frequencies. This new achievement in *A. thaliana* brings hope for future potential applications in other plant systems, some of which have been a subject of discussion over the past few years. In their review, Barrett et al. [115] discussed the necessity of developing synthetic gene drives to manage wild weed populations and inspected the factors that might affect these drives’ design, efficiency, and spread.

## 6. Conclusion

**Figure 2:**
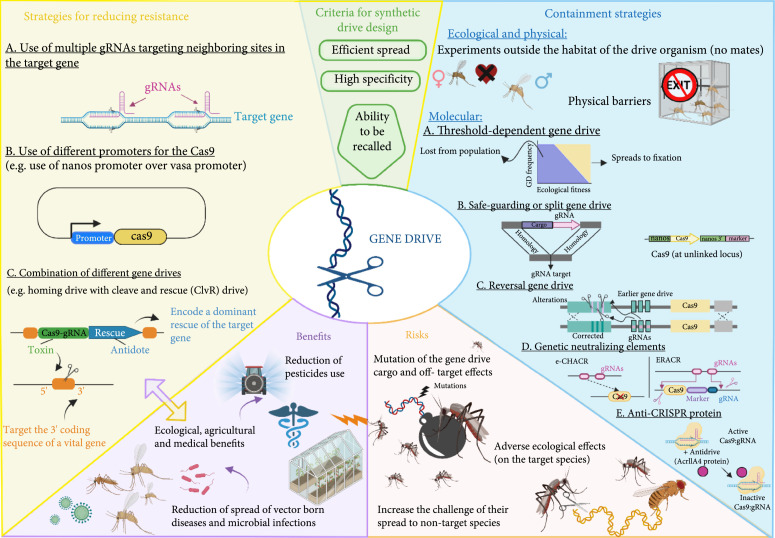
Designing gene drives to mitigate their unintended adverse risks and expand their potential benefits. All strategies have been tested experimentally but are still confined to laboratories and have not been established in the environment. A key challenge preventing the spread of gene drives and cutting back their benefits is the development of resistance. So far, three strategies have been published and shown a positive impact on reducing resistance allele formation in gene drives: (a) the multiplex gRNA expressing system [74, 75], (b) the use of the nanos promoter for Cas9 expression [75], and (c) the combination of different gene drives (e.g., homing endonuclease drive with cleave and rescue drive), showing a significant potential to overcome the accumulation of drive-resistant alleles [77, 81]. On the contrary, several containment strategies have been successfully proposed and established to mitigate the unintended negative impacts of gene drives on the environment. Among the molecular approaches: (a) a threshold-dependent gene drive [47], (b) a safeguarding gene drive, and (c) a reverse gene drive, were first designed and tested in *S. cerevisiae* [9]. (d) The use of genetic neutralizing elements [88] and (e) anti-Crispr protein [89] was also two novel containment measures in gene drive research.

To summarize, this review sheds light on the main benefits of gene drives and outlines critical considerations related to the risks of this double-edged tool. It also provides a comprehensive overview and discussion of the latest approaches developed in gene drive research to either improve implementation and utilization of its benefits or lessen its overall risks (Figure 2). Gene drive research has seen significant progress in recent years. Gene drive applications were developed in an incredible array of species, mainly after incorporating CRISPR tools, one of the biggest science stories over the last decade. However, the remaining open questions are as follows: what lies on the road ahead, and will gene drives bring the potential that they seem to hold? The answers to these questions are contingent upon many conditions and circumstances, the key one being agreement on a clearly defined scientific consensus on permissive conditions and risk assessments associated with the release of drive organisms into the wild, which is still a debate topic to this date.
